# DT-diaphorase activity in normal and neoplastic human tissues; an indicator for sensitivity to bioreductive agents?

**DOI:** 10.1038/bjc.1995.433

**Published:** 1995-10

**Authors:** E. Smitskamp-Wilms, G. Giaccone, H. M. Pinedo, B. F. van der Laan, G. J. Peters

**Affiliations:** Department of Oncology, Free University Hospital, Amsterdam, The Netherlands.

## Abstract

DT-diaphorase (DTD) is an important enzyme for the bioreductive activation of the new alkylating indoloquinone EO9. In preclinical studies, EO9 has shown selective anti-tumour activity against solid tumours and under hypoxic conditions. The levels of three reductive enzymes have been determined in three types of human solid tumours, together with corresponding normal tissues and normal liver. DTD enzyme activities were measured in tumour extracts using 2,6-dichlorophenolindophenol (DCPIP) and NADH as substrates; cytochrome P450 reductase or cytochrome b5 reductase activities were assessed with cytochrome c and NADPH or NADH respectively. DTD activity was highest in non-small-cell lung (NSCLC)-tumours (mean 123 nmol DCPIP min-1 mg-1), followed by colon carcinoma (mean 75 nmol min-1 mg-1) and squamous cell carcinoma of the head and neck (6-fold lower than NSCLC). DTD activity was very low in normal liver and normal lung (4-6 nmol min-1 mg-1), while the levels in normal colon mucosa or normal mucosa of the head and neck region were in the same range as the corresponding tumours. The levels of the two other reductive enzymes, cytochrome P450 reductase (CP450R) and cytochrome b5 reductase (Cb5R), were 5 to 25-fold lower than those of DTD in all the tissues, except for normal liver, in which DTD was 2 to 4-fold lower. The degree of variation found for DTD (range 4-250 nmol min-1 mg-1), was not observed for these enzymes (CP450R, 0.8-7.8 nmol cytochrome c min-1 mg-1; Cb5R, 3.5-27.6 nmol min-1 mg-1).(ABSTRACT TRUNCATED AT 250 WORDS)


British Journal of Cancer (1995) 72, 917-921
© 1995 Stockton Press All rights reserved 0007-0920/95 $12.00

### DT-diaphorase activity in normal and neoplastic human tissues; an indicator for sensitivity to bioreductive agents?

E Smitskamp-Wilms[1], G Giaccone[1], HM Pinedo[1], BFAM van der Laan[2] and GJ Peters[1]

[1]*Department of Oncology, Free University Hospital, PO Box 7057, 1007 MB Amsterdam, The Netherlands;* [2]*Department of Otolaryngology, University Hospital, Groningen, The Netherlands.*

**Summary** DT-diaphorase (DTD) is an important enzyme for the bioreductive activation of the new alkylating indoloquinone EO9. In preclinical studies, EO9 has shown selective anti-tumour activity against solid tumours and under hypoxic conditions. The levels of three reductive enzymes have been determined in three types of human solid tumours, together with corresponding normal tissues and normal liver. DTD enzyme activities were measured in tumour extracts using 2,6-dichlorophenolindophenol (DCPIP) and NADH as substrates; cytochrome P450 reductase or cytochrome b5 reductase activities were assessed with cytochrome *c* and NADPH or NADH respectively. DTD activity was highest in non-small-cell lung (NSCLC) tumours (mean 123 nmol DCPIP $min^{-1}$ $mg^{-1}$), followed by colon carcinoma (mean 75 nmol $min^{-1}$ $mg^{-1}$) and squamous cell carcinoma of the head and neck (6-fold lower than NSCLC). DTD activity was very low in normal liver and normal lung (4-6 nmol $min^{-1}$ $mg^{-1}$), while the levels in normal colon mucosa or normal mucosa of the head and neck region were in the same range as the corresponding tumours. The levels of the two other reductive enzymes, cytochrome P450 reductase (CP450R) and cytochrome b5 reductase (Cb5R), were 5 to 25-fold lower than those of DTD in all the tissues, except for normal liver, in which DTD was 2 to 4-fold lower. The degree of variation found for DTD (range 4-250 nmol $min^{-1}$ $mg^{-1}$), was not observed for these enzymes (CP450R, 0.8-7.8 nmol cytochrome *c* $min^{-1}$ $mg^{-1}$; Cb5R, 3.5-27.6 nmol $min^{-1}$ $mg^{-1}$). It is anticipated that NSCLC patients are more likely to respond to EO9 and other bioreductive agents, owing to the high levels of the activating enzyme in this tumour type. To prove that this assay has predictive value we need to study these enzyme levels in tumour samples obtained from patients on clinical studies with bioreductive agents.

**Keywords:** DT-diaphorase; head and neck squamous cell carcinoma; cytochrome b5 reductase; cytochrome P450 reductase; bioreductive agents; EO9.

DT-diaphorase (DTD, EC1.6.99.2) is considered to be the most important enzyme for the activation of bioreductive drugs such as EO9 and diaziquone (AZQ); at low pH it can also reduce mitomycin C (MMC) [Siegel *et al.*, 1990; Workman *et al.*, 1990; Walton *et al.*, 1991; Riley and Workman, 1992; Siegel *et al.*, 1992; Workman, 1992]. DTD is present in many mammalian tissues, where it can play a role in the biosynthesis of vitamin K (Wallin *et al.*, 1978; Ernster, 1987), or may act as a detoxifying enzyme, by catalysing a strict two-electron reduction (Benson *et al.*, 1980; Ernster, 1987). For AZQ and EO9 this reduction however leads to bioactivation of reactive groups, inducing their toxic properties such as DNA damage (Siegel *et al.*, 1990; Workman *et al.*, 1990; Walton *et al.*, 1991; 1992*a*). EO9, a bioreductive alkylating indoloquinone, is a structural analogue of MMC and has shown selective activity against solid tumour types, *in vitro* and in animal models (Oostveen and Speckamp, 1987; Hendriks *et al.*, 1993). Clinical phase I trials with EO9 have just been finished (Schellens *et al.*, 1994).

Several studies have been published recently, in which a correlation was postulated between the DTD levels (both expression and activity) and the chemosensitivity to EO9 under normoxic conditions (Robertson *et al.*, 1992; Walton *et al.*, 1992*b*; Plumb *et al.*, 1994*a*; Smitskamp-Wilms *et al.*, 1994). For other reductive enzymes such as the one-electron-donating NADH cytochrome b5 reductase (Cb5R) and NADPH cytochrome P450 reductase (CP450R) no such relation with EO9 sensitivity was found in more than 20 human tumour cell lines (Ross *et al.*, 1993; Plumb *et al.*, 1994*a*,*c*). It has however been reported that CP450R can reduce EO9 (Bailey *et al.*, 1994). Under hypoxic conditions the role of DTD is less clear; it seems that in cells with low DTD other reductases play a more prominent role under low-oxygen conditions, while metabolism of EO9 by DTD dominates in DTD-rich cells, in both air and hypoxia (Plumb *et al.*, 1994*a*,*b*,*c*; Robertson *et al.*, 1994). On the other hand, Yao *et al.* (1994) observed a 3- to 5-fold increase in DTD and CP450R mRNA by exposing a colon cell line to hypoxia. Apart from hypoxia, expression of DTD can also be induced by chemical inducers (Benson *et al.*, 1980; Prochaska and Talalay, 1988), including cytotoxic drugs or carcinogens. Several reports have mentioned higher DTD levels (both activity or mRNA) in tumour cells compared with normal tissues (Schor and Cornelisse, 1983; Schlager and Powis, 1990; Cresteil and Jaiswal, 1991), although not for all tumour types (Schlager and Powis, 1990).

The logical sequel to these studies would be to investigate the relation between DTD and sensitivity to bioreductive drugs in human tumour tissues. In this way it will be possible to select suitable tumours for treatment with, for example, EO9, following the 'enzyme directed approach' as put forward by Workman and Walton (1990). Therefore, we measured DTD levels in a number of human normal (from colon, lung, liver, blood and head and neck) and neoplastic tissues (from colon, lung and head and neck) to determine whether, and if so in which tumour types, EO9 would exert selectivity. The activity of two one-electron reductases, Cb5R and CP450R, was measured for comparison and because of their possible role in the activation of EO9 under hypoxia (Plumb *et al.*, 1994*b*,*c*; Robertson *et al.*, 1994).

### Materials and methods

### Patient characteristics

Biopsy specimens of primary colon tumours (CT, $n = 8$), metastases [Met, $n = 4$: three in liver (patients 10, 21 and 31) and one lymph node metastasis (patient 25)], normal mucosa (Muc, $n = 4$) or liver (Liv, $n = 6$) were obtained from 18 patients with colorectal cancer. Thirteen of them had received

Correspondence: GJ Peters
Received 26 January 1995; revised 15 May 1995; accepted 24 May 1995

one i.v. bolus injection of 5-fluorouracil (5FU; 500 mg $m^{-2}$) before surgery and have been described previously (Peters et al., 1993, 1994). The other five samples (CT 0'; Muc 3'; Liv 1', Liv 2' and Liv 3') were obtained from untreated patients (no chemotherapy at the time of surgery). From several patients both tumour and normal tissues were acquired (samples with the same number).

The second group consisted of eight patients suffering from squamous cell carcinoma (SCC) of the head and neck region; three oropharynx, two hypopharynx, one supraglottic, two floor of the mouth. Five of them had received radiotherapy before surgery. From all of these patients, tumour tissue was removed together with adjacent intermediate tissue and normal appearing tissue, which were stored separately. Strict non-neoplastic tissues from the head and neck region (uvulae) were obtained from non-cancer patients undergoing reconstructive palato-pharyngeal surgery. These samples were treated in the same way as the tumours.

All the lung specimens were obtained from patients with non-small-cell lung cancer (NSCLC) undergoing radical resection. Four tumours were classified as squamous cell carcinoma (SCC), one as an adenocarcinoma (AD), one as combined adenosquamous (AS) and one as large cell (LC). All patients had a smoking history.

Blood was taken from a healthy volunteer. After centrifugation, the fraction with the red cells was sonicated on ice.

*Sample preparation*

Biopsy specimens were obtained from several tumours and histologically confirmed. Adjacent normal appearing tissue was frozen separately. Normal and tumour tissues had been frozen immediately after surgery in liquid nitrogen and kept at −80°C. At the day of the enzyme assays, the still frozen tumour was cut into small pieces (with a total weight of maximum ± 100 mg) and was pulverised in a liquid nitrogen-cooled vessel using a microdismembrator (B Braun, Melsungen, Germany, Peters et al., 1986). Thus during the whole procedure the tumour was kept frozen. The powder was homogenised by vortexing in ice-cold Tris-HCl buffer (25 mM, pH 7.4) at 1 g of tissue per 6 ml of buffer and centrifuged for 5 min at 4000 r.p.m. at 4°C. After removal of the fatty layer, the supernatant was subsequently centrifuged for 15 min at 14 000 r.p.m. at 4°C. The second supernatant was used for enzyme measurements. If necessary, it was diluted 5–10 times in 25 mM Tris buffer. A small sample of the enzyme extract was frozen again in liquid nitrogen and kept for several months at −80°C to test stability. DTD activity was stable for at least 6 months (results not shown). Reproducibility was tested by extracting samples of one biopsy specimen at different time points. Inter- and intra-assay variation was less than 10%. Protein concentrations were determined according to Bradford (1976).

*Bioreductive enzyme assays*

DTD was measured spectrophotometrically as described earlier for cell lines, except that addition of bovine serum albumin (BSA) as activator or stabiliser was not necessary (Smitskamp-Wilms et al., 1994). Briefly, the reaction was performed in a total volume of 3 ml, containing 25 mM Tris pH 7.4; 40 μM dichlorophenolindophenol (DCPIP) as substrate and 0.2 mM NADH as co-factor (final concentrations). The reduction was measured at room temperature for 1–3 min at 600 nm with or without the inhibitor dicoumarol (100 μM). DTD activity is considered to be the dicoumarol-inhibitable part of the DCPIP reduction. The reaction was started by addition of at least three different volumes of the supernatant (0.2–50 μl 3 $ml^{-1}$), which enabled us to study linearity. When measuring DTD activity in the blood samples, the same volume of sonicated red blood cells was added to both cuvettes, to compensate for the high absorbance caused by the haemoglobin.

NADH cytochrome b5 reductase and NADPH cytochrome P450 reductase activity were measured at 550 nm under similar conditions, using as a substrate 77 μM cytochrome *c* and as co-factors 0.2 mM NADH or 0.2 mM NADPH respectively. Cytochrome *c* is not a substrate for DTD, while DTD has no preference for either of the co-factors (Ernster et al., 1962). Units of activity were expressed as nmol DCPIP reduced $min^{-1}$ $mg^{-1}$ protein for DTD and as nmol cytochrome *c* reduced $min^{-1}$ $mg^{-1}$ protein for NADH cytochrome b5 reductase and NADPH cytochrome P450 reductase.

Control experiments concerning induction of DTD by 5FU or EO9 were performed on the colon carcinoma cells lines SW620 and WiDr. DTD activity was determined as described previously (Smitskamp-Wilms et al., 1994) 24 h after exposure to 25 μM 5FU or 30 nM EO9 ($IC_{50}$ values). 5FU concentrations in the colon tumours varied from 0.17–21.6 μM (Peters et al., 1993).

*Statistical evaluation*

Statistical evaluation of the differences between the samples was tested using the Mann–Whitney *U*-rank-sum test.

### Results

Measurement of DTD activity in tumour tissues that has been stored frozen, is feasible. To assay DTD no more than 60 mg of frozen tissue is required compared with $10^5$ cells, when cell lines were used (Smitskamp-Wilms et al., 1994). The DTD activity in the different tissue types are depicted in Figure 1a, b and c. As a group DTD activity was highest in NSCLC tumours, followed by colon tumours and metastases (three liver and one lymph node) of colon tumours. Normal lung and liver tissue and squamous cell carcinoma of the head and neck contained low DTD activity (Table I). In the sets of paired tissues obtained from three NSCLC patients, all tumours clearly had significantly elevated DTD levels ($P = 0.008$) compared with normal lung tissues (7-, 12- and 66-fold higher; Figure 1c). In colon mucosa, this difference was less clear. From the three paired samples, the DTD activity was three times lower in one mucosa than in the corresponding tumour (patient 7), while in the other two patients (patients 19 and 55), there was no difference in DTD activity (Figure 1a). The colon tumours showed a varying range of DTD activities. In four of the primary colon tumours, DTD activity was higher than 46 nmol $min^{-1}$ $mg^{-1}$. In the other four, in the colon cancer metastases (three liver and one lymph node) and in normal colon mucosa, intermediate levels were found (2–31 nmol $min^{-1}$ $mg^{-1}$), while in normal liver it was very low (4 nmol $min^{-1}$ $mg^{-1}$). This means that the metastatic tissue is more similar to the primary tumour than to the local liver tissue (Figure 1a).

Thirteen of the patients in the colon cancer group had received a single injection of 5FU just before the surgery. However, DTD activities in the three livers from treated patients (1', 2', 3') were not different from the three control (no 5FU) livers (patients 17, 57, 58). Similarly, other samples from untreated patients (primary tumour and mucosa) had DTD levels in the same range as that of treated patients. As a control, possible induction by 5FU or EO9 was measured in two colon carcinoma cell lines. Both in SW620 and WiDr cells, no induction by these compounds was observed after 24 h exposure to $IC_{50}$ concentrations (results not shown).

In the head and neck tumours we observed low to intermediate DTD levels. In these tumours the activity was much lower than in the lung and colon tumours; in addition the activity was not significantly different from normal (Mann–Whitney *U*-test: $P = 0.4$) or intermediate ($P = 0.14$) tissues, including normal tissue from non-cancer patients (Table 1, Figure 1b).

In normal liver, DTD activity measured as DCPIP reduction was very low (4 nmol $min^{-1}$ $mg^{-1}$). Whereas in the tumour extracts low concentrations of dicoumarol almost completely inhibited the enzyme activities (>90%) similar to cell lines, in normal liver dicoumarol inhibited only 40–50%

**Figure 1** DTD levels in human tissues. Indicated is the site of the sample and the type of tissue (■, tumour; ■, normal tissue), followed by a patient's number (abbreviations used as described in Materials and methods). (**a**) CT, colon tumour; Muc, normal colon mucosa; Liv, normal liver; Met, metastasis, from primary colon cancer; Patients' numbers followed by ' did not receive a 5FU injection; (**b**) HN, head and neck; (**c**) L, NSCLC patients.

**Table I** DTD activities in human tumour and normal tissues

| *Origin* | *Tissue type* | *DTD*[a] *mean* ± *s.d.* | *Median* | *Range* | n[b] |
|---|---|---|---|---|---|
| Colon | Primary tumour | 75* ± 87 | 48 | 10–290 | 8 |
| | Normal mucosa | 30 ± 20 | 25 | 13–55 | 4 |
| | Lymph node Metastasis | 18 | | | 1 |
| | Liver metastasis | 17.4* ± 12.1 | 20 | 1–31 | 3 |
| Liver | Normal | 3.7 ± 0.7 | 3.9 | 3–5 | 6 |
| Lung | NSCLC | 123** ± 74 | 138 | 16–250 | 7 |
| | Normal | 6.4 ± 4 | 6.9 | 2–10 | 3 |
| Head and neck | SSC tumour | 21* ± 12.6 | 21 | 4–34 | 8 |
| | Intermediate | 30.7* ± 14.9 | 27.4 | 10–50 | 8 |
| | Normal | 19.5 ± 7.2 | 19.4 | 7–29 | 8 |
| | Uvulae[c] | 57* ± 34 | 54 | 14–100 | 3 |

[a]DTD activity: mean ± standard deviation (s.d.), unit: nmol $min^{-1}$ $mg^{-1}$. [b]$n$ = number of individual samples (biopsy specimen). [c]Normal tissue from non-cancer patients. *Not significantly different from corresponding normal tissue ($P > 0.05$). **Significantly different from normal tissue ($P = 0.008$). The Mann–Whitney $U$-test was employed for statistical analysis. Level of significance is set at $P < 0.05$.

of the reduction of DCPIP. This indicates the presence of other enzymes that are able to reduce DCPIP. This was confirmed by measuring the activity of Cb5R and CP450R (Figure 2). In all tested tumours and colon mucosa these one-electron-donating enzymes were less abundant than DTD (Table II), although it is difficult to draw an exact parallel since substrates differ. Levels varied between 5% and 60% of that of DTD (range 3.5–27.6 nmol $min^{-1}$ $mg^{-1}$, median 13 nmol $min^{-1}$ $mg^{-1}$). In contrast, the liver extracts contained much higher Cb5R activity compared with that of DTD (230–470%), although the absolute activities were in the same range (7.6–23 nmol $min^{-1}$ $mg^{-1}$) as in the colon tissues. Also, absolute CP450R activities were similar among liver and other tissues (range 0.8–7.8 nmol $min^{-1}$ $mg^{-1}$). But, relative to DTD, in normal liver CP450R activities were higher than in other tissues.

DTD could not be detected in red blood cells. Although DCPIP was being reduced at a relatively low level (5 nmol $min^{-1}$ $mg^{-1}$ protein or 300 nmol $min^{-1}$ $ml^{-1}$ red blood cells), this reduction could not be inhibited by dicoumarol.

Similar to the DTD activity in cell lines (Smitskamp-Wilms *et al.*, 1994), we noticed variations in the linearity of the assay depending on the source of tissue. For some of the samples with high DTD activity (> 50 nmol $min^{-1}$ $mg^{-1}$ or > 250 nmol $min^{-1}$ $ml^{-1}$), it was necessary to use higher dilutions in order to perform measurements in the linear range. This range varied per sample but in general was in the low protein range (< 60 μg per assay) for samples with high activity (> 50 nmol $min^{-1}$ $mg^{-1}$). For tissues with an activity lower than 25 nmol $min^{-1}$ $mg^{-1}$ (or < 100 nmol $min^{-1}$ $ml^{-1}$), this range was less narrow and went up to 300 μg per assay.

### Discussion

In this study we demonstrate that only in human lung tumours (NSCLC) DTD activity was significantly higher than in normal tissues. In the other tumour types investigated (colon, including some metastases, and SSC of the head and neck) this difference was not observed, whereas the

**Figure 2** One-electron reductase (▒, NADH cytochrome b5 reductase; ■, NADPH cytochrome P450 reductase) activity in human tissues. The sample codes are the same as used in Figure 1. SQ, squamous cell carcinoma; AS, combined adenosquamous; AD, adenocarcinoma; LC, large cell.

DTD activity in these tumours was also lower than in lung tumours.

These high levels in NSCLC samples are in agreement with the data reported by Malkinson *et al.* (1992); Ross *et al.* (1993). Much higher DTD levels were found in NSCLC compared with small-cell lung cancer and normal lung. Generally, DTD levels in cell lines derived from NSCLC were higher than in small-cell lung cancer (SCLC) cell lines or other cell lines (Malkinson *et al.*, 1992; Robertson *et al.*, 1992, 1994). These results indicate that NSCLC is more likely to be sensitive to EO9 than other tumour types (Ross *et al.*, 1993; Stratford *et al.*, 1994). Consequently, NSCLC is one of the diseases selected for phase II clinical trials.

Several reports mentioned that the activity of DTD is higher in tumour cells than in normal cells of the same origin (Schor and Cornelisse 1983; Schlager and Powis 1990; Cresteil and Jaiswal 1991; Malkinson et al., 1992). This has been found in primary tumours of lung, liver, colon, breast and testis (Schlager and Powis, 1990). However, in tumours from kidney and stomach, lower enzyme activities were found when compared with normal tissue (Schlager and Powis 1990; Eickelmann *et al.*, 1994). In this study, in addition to small differences between normal colon mucosa and primary colon tumours, we found that head and neck tumours showed the same activity pattern as the surrounding normal tissues. Normal tissue from the head and neck region from non-cancer patients showed the same DTD levels, indicating that the results were not influenced by their location with respect to the tumour.

Studies on regulation of expression of DTD (Prochaska and Talalay 1988; Cresteil and Jaswail 1991: Belinsky and Jaiswal 1993) indicated that DTD can be induced by cytotoxic drugs. However, our results show no difference in DTD activities between three livers from patients who had

**Table II** Reductive enzymes in a set of human tissues (subset of the samples mentioned in Table 1)

| | Reductive enzymes | | | |
|---|---|---|---|---|
| *Tumour type* | *DTD*[a] *nmol DCPIP min*$^{-1}$ *mg*$^{-1}$ | *Cb5R*[a] *nmol Cyt* c *min*$^{-1}$ *mg*$^{-1}$ | *CP450R*[b] *nmol Cyt* c *min*$^{-1}$ *mg*$^{-1}$ | n |
| Normal liver | 3.9 | 15.5 | 6.7 | (5) |
| NSCLC | 138 | 13.3 | 5.1[b] | (4) |
| Colon tumour | 119 | 17.8 | 3.6[b] | (4) |
| Colon mucosa | 35.6 | 6.9 | 5.7 | (3) |

[a]Values are means of *n* samples (as indicated within parentheses), [b]except for CP450R in NSCLC and colon tumours where $n = 1$. Intra-assay variation among duplicates was <10%.

been treated with 5FU and three liver samples from untreated patients (LIV 1′, 2′ and 3′). Also *in vitro* no induction by physiologically relevant concentrations of 5FU or EO9 was observed.

The very low DTD activities in human normal liver are in sharp contrast to DTD in rodent normal livers (Schlager and Powis 1990). Differences in kinetic behaviour of isolated rat and human DTD as described by Boland *et al.* (1991) emphasise again that rodent models are not always appropriate models for human drug therapy.

One-electron reductase activities in tumour tissues were 2–60% compared with that of DTD, while cytochrome b5 reductase levels were always higher than those of cytochrome P450 reductase. This was in line with other studies, reporting levels varying around 20% of that of DTD (Schlager and Powis 1990; Walton *et al.*, 1992; Hendriks *et al.*, 1993; Ross *et al.*, 1993). Relatively high levels of these enzymes contribute to the reduction of DCPIP in the DTD assay, as was noticed in normal liver. In these samples, total reductase activity on DCPIP could be inhibited only by 50% by dicoumarol. Although no correlation between the levels of these enzymes (measured under aerobic conditions) and EO9 cytotoxicity in cell lines was found, it is conceivable that these enzymes play a role in the hypoxic part of the tumour (Plumb *et al.*, 1994*a*,*c*). However, the small differences among the several tissue types rule out a possible role as predictive marker for these enzymes.

The potency of EO9 under hypoxic conditions (Adams *et al.*, 1992) makes it an interesting compound to combine with radiation. Since encouraging results have been found in preclinical studies using cell lines and rats, clinical trials with this combination should be considered (Adams and Stratford 1994; Kal *et al.*, 1994; Stratford *et al.*, 1994).

Correlations between EO9 sensitivity and DTD activity *in vivo* are limited to only a few murine models (Workman *et al.*, 1990; Walton *et al.*, 1992) and rat (Kal *et al.*, 1994). Although the relation was positively similar to that found in *in vitro* studies, DTD levels in patients entering clinical trials should be measured since these levels are expected to have predictive value. High DTD levels would be associated with a response to EO9. This is in line with the theory of enzyme profiling before determining therapy, as suggested by Workman and Walton (1990; Riley and Workman 1992; Walton *et al.*, 1992*b*).

In conclusion, based on these observations in human tumours (absolute activity and relative to normal tissue), patients with NSCLC and those with colon tumours are more likely to be sensitive to EO9 than those with squamous cell carcinoma of head and neck. This should be validated by measuring DTD levels in patients undergoing EO9 treatment, such as those participating in the clinical trials.

### References

ADAMS GE, STRATFORD IJ, EDWARDS HS, BREMNER JCM AND COLE S. (1992). Bioreductive drugs as post-irradiation sensitizers: comparison of dual function agents with SR4233 and the Mitomycin C analogue EO9. *Int. J. Radiat. Oncol. Biol. Phys.*, **22**, 717–720.

ADAMS GE AND STRATFORD IJ. (1994). Bioreductive drugs for cancer therapy: The search for tumor specificity. *Int. J. Radiat. Oncol. Biol. Phys.*, **29**, 231–238.

BAILEY SM, LEWIS AD AND WORKMAN P. (1994). Involvement of NADPH: Cytochrome P450 reductase in activation of EO9. *Br. J. Cancer*, **69**, (suppl. XXI) 57.

BELINSKY M AND JAISWAL AK. (1993). NAD(P)H-Quinone oxidoreductase(1) (DT-diaphorase) expression in normal and tumor tissues. *Cancer Metastasis Rev.*, **12**, 103-117.

BENSON AN, HUNKELER MJ AND TALALAY P. (1980). Increase of NAD(P)H:quinone reductase by dietary antioxidants: Possible role in protection against carcinogenesis and toxicity. *Proc. Natl Acad. Sci. USA*, **77**, 5216-5220.

BOLAND MP, KNOX RJ AND ROBERTS JJ. (1991). The differences in kinetics of rat and human DT-diaphorase result in a differential sensitivity of derived cell lines to CB1954 (5-(aziridin-1-yl)-2, 4-dinitrobenzamide). *Biochem. Pharmacol.*, **41**, 867-875.

BRADFORD MM. (1976). A rapid and sensitive method for the quantification of microgram quantities of protein utilizing the principle of protein-dye binding. *Anal. Biochem.*, **72**, 248-254.

CRESTEIL T AND JAISWAL AK. (1991). High levels of expression of the NAD(P)H:quinone oxidoreductase (NQ01) gene in tumor cells compared to normal cells of the same origin. *Biochem. Pharmacol.*, **42**, 1021-1027.

EICKELMANN P, EBERT T, WARSKULAT U, SCHULZ WA AND SIES H. (1994). Expression of NAD(P)H:quinone Oxidoreductase and Glutathione S-Transferases alpha and pi in Human Renal Cell Carcinoma and in Kidney Cancer-Derived Cell Lines. *Carcinogenesis*, **15**, 219-225.

ERNSTER L. (1987). DT diaphorase: A historical review. *Chemica Scripta*, **27A**, 1-13.

ERNSTER L, DANIELSON L AND LJUNGGREN M. (1962). DT diaphorase I. Purification from the soluble fraction of rat-liver cytoplasm, and properties. *Biochim. Biophys. Acta*, **58**, 171-188.

HENDRIKS HR, PIZAO PE, BERGER DP, KOOISTRA KL, BIBBY MC AND BOVEN E. (1993). EO9: a novel bioreductive alkylating indoloquinone with preferential solid tumour activity and lack of bone marrow toxicity in preclinical models. *Eur. J. Cancer*, **29A**, 897-906.

KAL HB, KARIM ABMF AND HENDRIKS HR. (1994). The efficacy of EO9 with radiation in experimental rat tumors. *Ann. Oncol.*, suppl. 5, 88.

MALKINSON AM, SIEGEL D, FORREST GL, GAZDAR AF, OIE HK, CHAN DC, BUNN PA, MABRY M, DYKES DJ, HARRISON SD AND ROSS D. (1992). Elevated DT-diaphorase activity and messenger RNA content in human non-small cell lung carcinoma-relationship to the response of lung tumor xenografts to mitomycin C. *Cancer Res.*, **52**, 4752-4757.

OOSTVEEN EA AND SPECKAMP WN. (1987). Mitomycin analogues 1. Indoloquinones as potent bisalkylating agents. *Tetrahedron*, **43**, 255-262.

PETERS GJ, LAURENSSE E, LEYVA A AND PINEDO HM. (1986). Tissue homogenization using a microdismembrator for the measurement of enzyme activities. *Clin. Chim. Acta*, **158**, 193-198.

PETERS GJ, LANKELMA J, KOK RM, NOORDHUIS P, VAN GROENINGEN CJ, VAN DER WILT CL, MEIJER S AND PINEDO HM. (1993). Prolonged retention of high concentrations of 5-fluorouracil in human and murine tumors as compared with plasma. *Cancer Chemother. Pharmacol.*, **31**, 269-276.

PETERS GJ, VAN DER WILT CL, VAN GROENINGEN CJ, SMID K, MEIJER S AND PINEDO HM. (1994). Thymidylate synthase inhibition after administration of 5-Fluorouracil with or without leucovorin in colon cancer patients; implications for treatment with 5-Fluorouracil. *J. Clin. Oncol.*, **12**, 2035-2042.

PLUMB JA, GERRITSEN M, MILROY R, THOMSON P AND WORKMAN P. (1994*a*). Relative importance of DT-diaphorase and hypoxia in the bioactivation of EO9 by human lung tumor cell lines. *Int. J. Radiat. Oncol. Biol. Phys.*, **29**, 295-299.

PLUMB JA AND WORKMAN P. (1994*b*). Unusually marked hypoxic sensitization to indoloquinone EO9 and mitomycin C in a human colon-tumour cell line that lacks DT-diaphorase activity. *Int. J. Cancer*, **56**, 134-139.

PLUMB JA, GERRITSEN M AND WORKMAN P. (1994*c*). DT-diaphorase protects cells from the hypoxic toxicity of indoloquinone EO9. *Br. J. Cancer*, **70**, 1136-1143.

PROCHASKA HJ AND TALALAY P. (1988). Regulatory mechanisms of monofunctional and bifunctional anticarcinogenic enzyme inducers in murine livers. *Cancer Res.*, **48**, 4776-4782.

RILEY RJ AND WORKMAN P. (1992). DT-diaphorase and cancer chemotherapy. *Biochem. Pharmacol.*, **43**, 1657-1669.

ROBERTSON N, STRATFORD IJ, HOULBROOK S, CARMICHAEL J AND ADAMS GE. (1992). The sensitivity of human tumour cells to quinone bioreductive drugs: What role for DT-diaphorase? *Biochem. Pharmacol.*, **44**, 409-412.

ROBERTSON N, HAIGH A, ADAMS GE AND STRATFORD IJ. (1994). Factors affecting sensitivity to EO9 in rodent and human tumour cells in vitro: DT-diaphorase activity and hypoxia. *Eur. J. Cancer*, **30a**, 1013-1019.

ROSS D, SIEGEL D, BEALL H, PRAKASH AS, MULCAHY RT AND GIBSON NW. (1993). DT-diaphorase in activation and detoxification of quinones - bioreductive activation of mitomycin-C. *Cancer Metastasis Rev.*, **12**, 83-101.

SCHELLENS JHM, PLANTING AST, VAN ACKER BAC, LOOS WJ, DE BOER-DENNERT M, VAN DER BURG MEL, KOIER I, KREDIET RT, STOTER G AND VERWEIJ J. (1994). Phase I and pharmacologic study of the novel indoloquinone bioreductive alkylating cytotoxic drug EO9. *J. Natl Cancer Inst.*, **86**, 906-912.

SCHLAGER JJ AND POWIS G. (1990). Cytosolic NAD(P)H:(quinone-acceptor)oxidoreductase in human normal and tumor tissue: effects of cigarette smoking and alcohol. *Int. J. Cancer*, **45**, 403-409.

SCHOR NA AND CORNELISSE CJ. (1983). Biochemical and quantitative histochemical study of reduced pyridine nucleotide dehydrogenation by human colon carcinomas. *Cancer Res.*, **43**, 4850-4855.

SIEGEL D, GIBSON NW, PREUSCH PC AND ROSS D. (1990). Metabolism of Diaziquone by NAD(P)H:(Quinone acceptor) oxidoreductase: role in Diaziquone-induced DNA damage and cytotoxicity in human colon carcinoma cells. *Cancer Res.*, **50**, 7293-7300.

SIEGEL D, BEALL H, SENEKOWITSCH C, KASAI M, ARAI H, GIBSON NW AND ROSS D. (1992). Bioreductive Activation of Mitomycin C by DT-Diaphorase. *Biochem.*, **31**, 7879-7885.

SMITSKAMP-WILMS E, PETERS GJ, PINEDO HM, VAN ARK-OTTE J. AND GIACCONE G. (1994). Chemosensitivity to the indoloquinone EO9 is correlated with DT-diaphorase activity and its gene expression. *Biochem. Pharmacol.*, **47**, 1325-1332.

STRATFORD IJ, ADAMS GE, BREMNER JCM, COLE S, EDWARDS HS, ROBERTSON N AND WOOD PJ. (1994). Manipulation and exploitation of the tumour environment for therapeutic benefit. *Int. J. Radiat. Biol.*, **65**, 85-94.

WALLIN R, GEBHARDT O AND PRYDZ H. (1978). NAD(P)H dehydrogenase and its role in vitamin K (2-methyl-3-phytyl-1,4-naphthaquinone)-dependent carboxylation reaction. *Biochem. J.*, **169**, 95-101.

WALTON MI, SMITH PJ AND WORKMAN P. (1991). The role of NAD(P)H:quinone reductase (EC 1.6.99.2, DT-diaphorase) in the reductive bioactivation of the novel indoloquinone antitumor agent EO9. *Cancer Commun.*, **3**, 199-206.

WALTON MI, BIBBY MC, DOUBLE JA, PLUMB JA AND WORKMAN P. (1992*a*). DT-diaphorase activity correlates with sensitivity to the indoloquinone-EO9 in mouse and human colon carcinomas. *Eur. J. Cancer*, **28A**, 1597-1600.

WALTON MI, SUGGET N AND WORKMAN P. (1992*b*). The role of human and rodent DT-diaphorase in the reductive metabolism of hypoxic cell cytotoxins. *Int. J. Radiat. Oncol. Biol. Phys.*, **22**, 643-647.

WORKMAN P AND WALTON MI. (1990). Enzyme directed bioreductive drug development. In *Selective Activation of Drugs by Redox Processes*. Adams GE, Breccia A, Fielden EM and Wardman P. (eds) pp. 173-191. Plenum Press: New York.

WORKMAN P, WALTON MI, BIBBY MC AND DOUBLE JA. (1990). In vivo response of mouse adenocarcinoma of the colon (MAC) tumours to indoloquinone EO9: correlation with bioreductive enzyme content. *Br. J. Cancer*, **62**, 515-516.

WORKMAN P. (1992). Keynote Address: Bioreductive mechanisms. *Int. J. Radiat. Oncol. Biol. Phys.*, **22**, 631-637.

WORKMAN P, BINGER M AND KOOISTRA K. (1992). Pharmacokinetics, distribution and metabolism of the novel bioreductive alkylating indoloquinone EO9 in rodents. *Int. J. Radiat. Oncol. Biol. Phys.*, **22**, 713-716.

YAO KS, CLAYTON M AND O'DWYER PJ. (1994). Interaction of heat and hypoxia in modulating transcription of DT-diaphorase in human colon adencarcinoma cells. *Cell Growth Differ.*, **5**, 125-131.


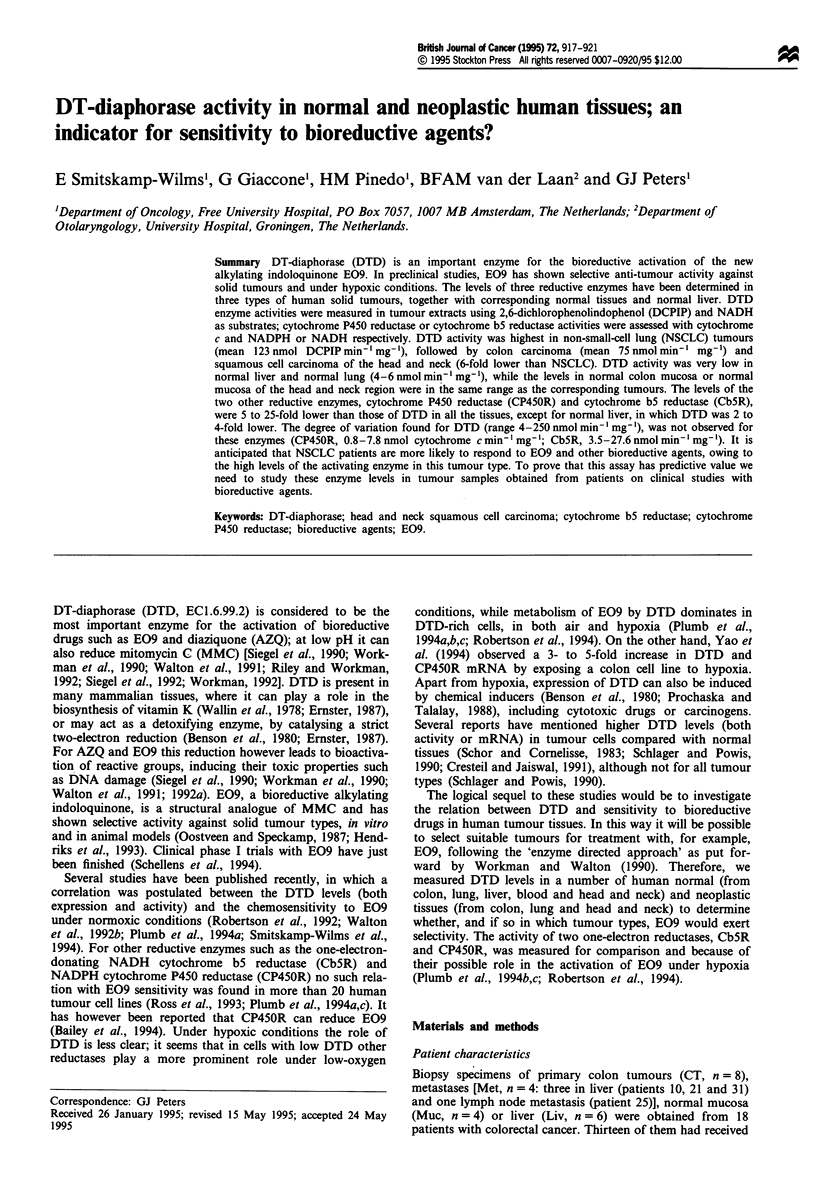

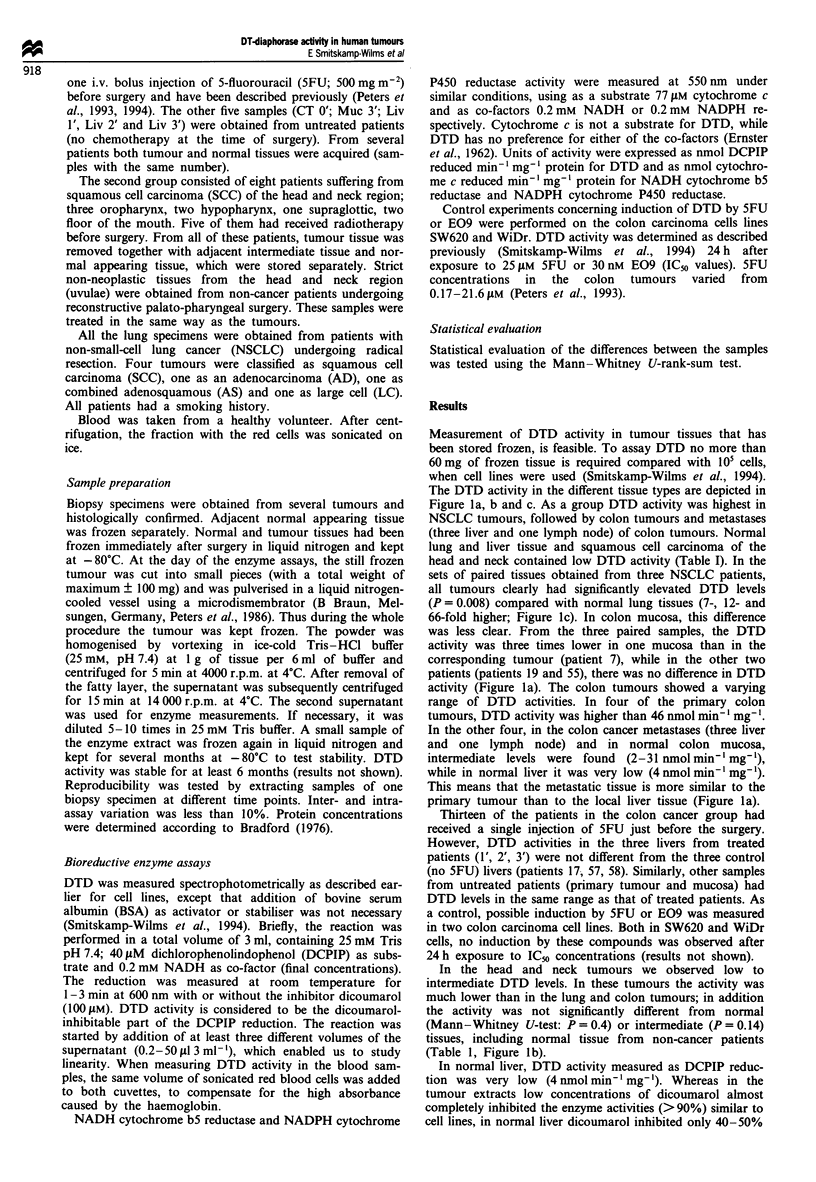

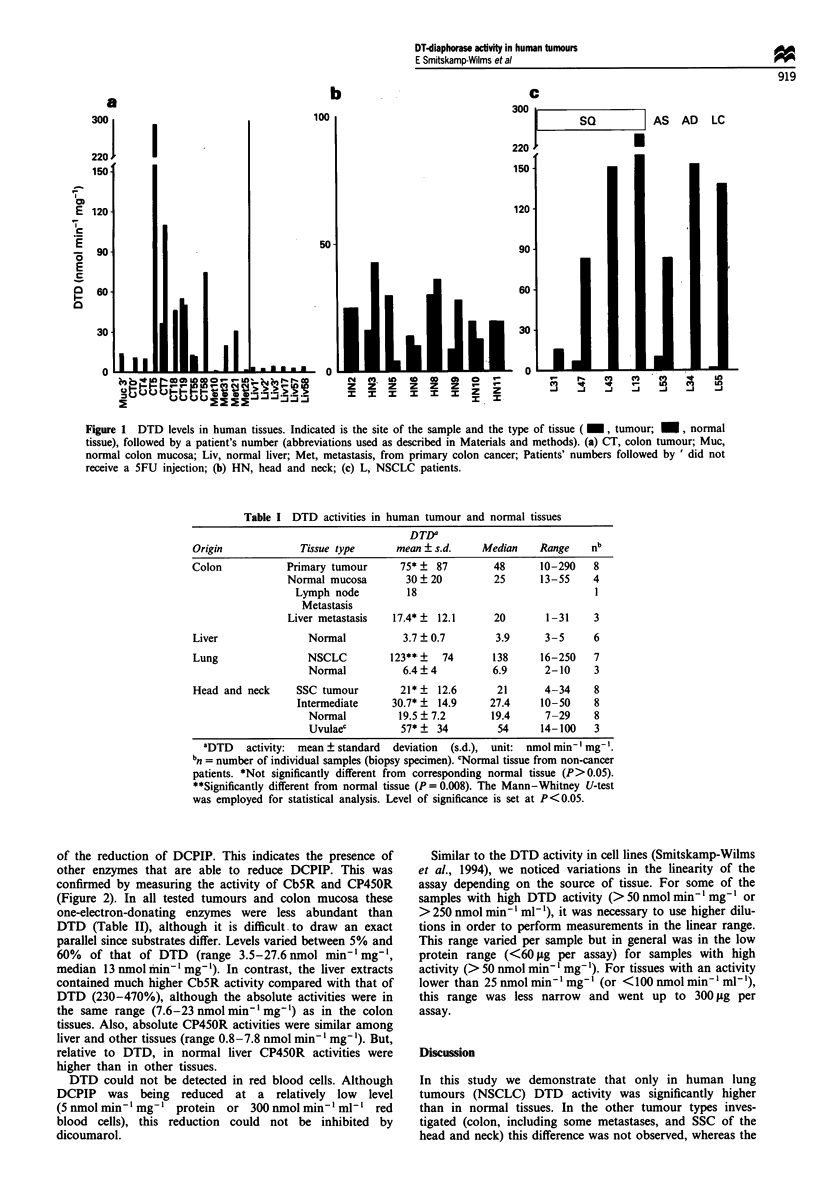

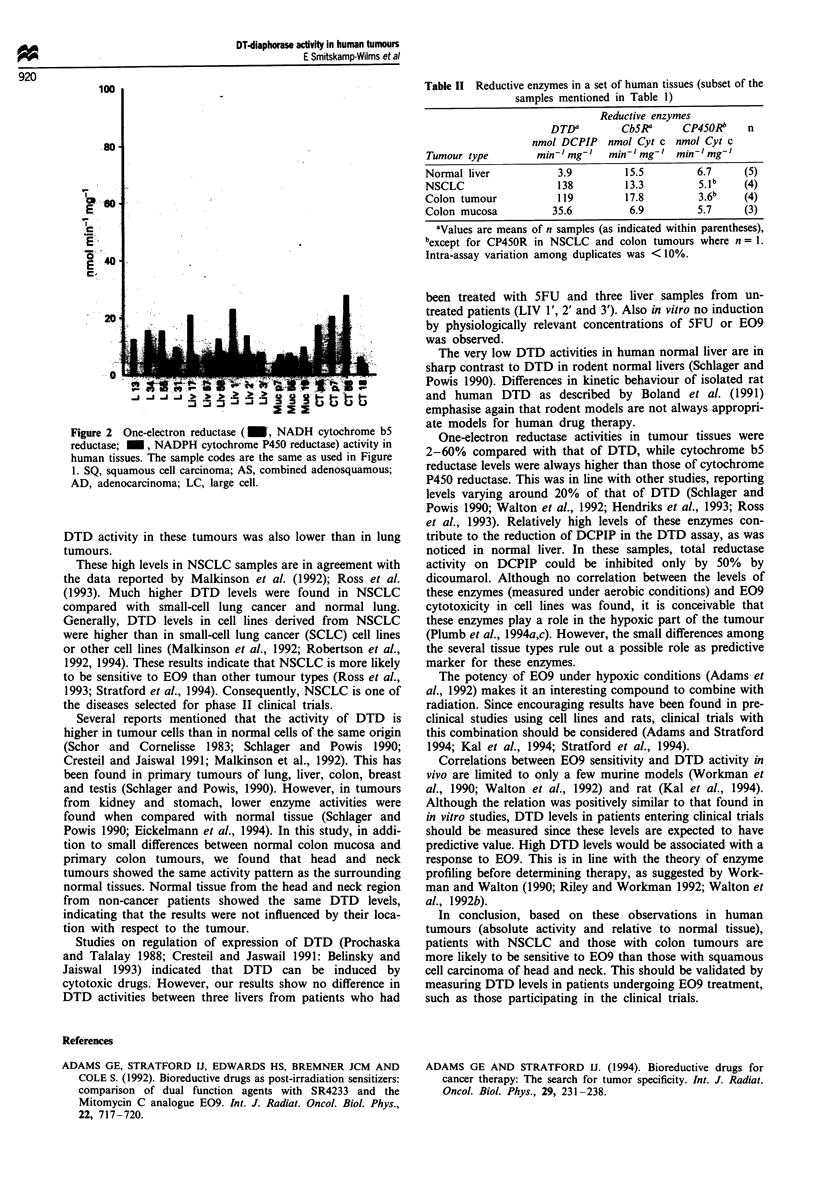

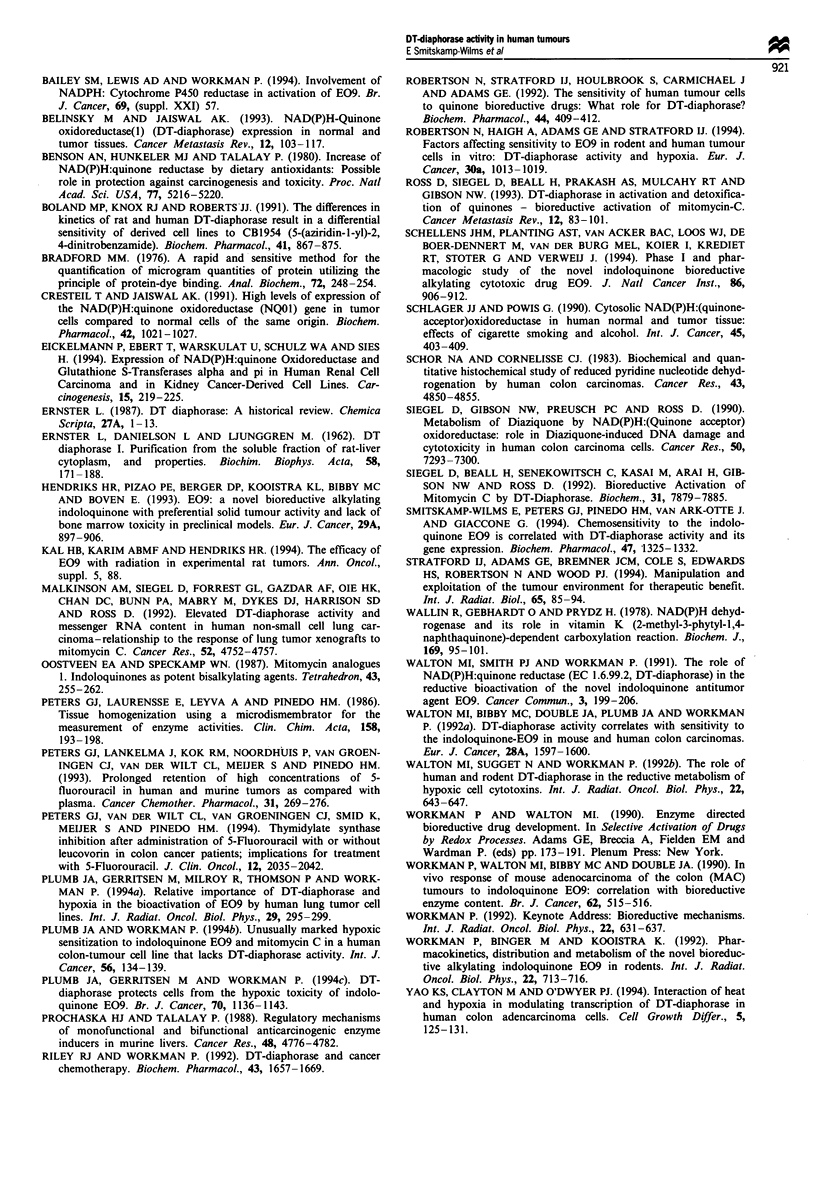


## References

[OCR_00574] Adams G. E., Stratford I. J. (1994). Bioreductive drugs for cancer therapy: the search for tumor specificity.. Int J Radiat Oncol Biol Phys.

[OCR_00567] Adams G. E., Stratford I. J., Edwards H. S., Bremner J. C., Cole S. (1992). Bioreductive drugs as post-irradiation sensitizers: comparison of dual function agents with SR 4233 and the mitomycin C analogue EO9.. Int J Radiat Oncol Biol Phys.

[OCR_00592] Belinsky M., Jaiswal A. K. (1993). NAD(P)H:quinone oxidoreductase1 (DT-diaphorase) expression in normal and tumor tissues.. Cancer Metastasis Rev.

[OCR_00597] Benson A. M., Hunkeler M. J., Talalay P. (1980). Increase of NAD(P)H:quinone reductase by dietary antioxidants: possible role in protection against carcinogenesis and toxicity.. Proc Natl Acad Sci U S A.

[OCR_00603] Boland M. P., Knox R. J., Roberts J. J. The differences in kinetics of rat and human DT diaphorase result in a differential sensitivity of derived cell lines to CB 1954 (5-(aziridin-1-yl)-2,4-dinitrobenzamide). Biochem Pharmacol.

[OCR_00609] Bradford M. M. (1976). A rapid and sensitive method for the quantitation of microgram quantities of protein utilizing the principle of protein-dye binding.. Anal Biochem.

[OCR_00614] Cresteil T., Jaiswal A. K. (1991). High levels of expression of the NAD(P)H:quinone oxidoreductase (NQO1) gene in tumor cells compared to normal cells of the same origin.. Biochem Pharmacol.

[OCR_00631] ERNSTER L., DANIELSON L., LJUNGGREN M. (1962). DT diaphorase. I. Purification from the soluble fraction of rat-liver cytoplasm, and properties.. Biochim Biophys Acta.

[OCR_00620] Eickelmann P., Ebert T., Warskulat U., Schulz W. A., Sies H. (1994). Expression of NAD(P)H:quinone oxidoreductase and glutathione S-transferases alpha and pi in human renal cell carcinoma and in kidney cancer-derived cell lines.. Carcinogenesis.

[OCR_00638] Hendriks H. R., Pizao P. E., Berger D. P., Kooistra K. L., Bibby M. C., Boven E., Dreef-van der Meulen H. C., Henrar R. E., Fiebig H. H., Double J. A. (1993). EO9: a novel bioreductive alkylating indoloquinone with preferential solid tumour activity and lack of bone marrow toxicity in preclinical models.. Eur J Cancer.

[OCR_00650] Malkinson A. M., Siegel D., Forrest G. L., Gazdar A. F., Oie H. K., Chan D. C., Bunn P. A., Mabry M., Dykes D. J., Harrison S. D. (1992). Elevated DT-diaphorase activity and messenger RNA content in human non-small cell lung carcinoma: relationship to the response of lung tumor xenografts to mitomycin Cł.. Cancer Res.

[OCR_00666] Peters G. J., Lankelma J., Kok R. M., Noordhuis P., van Groeningen C. J., van der Wilt C. L., Meyer S., Pinedo H. M. (1993). Prolonged retention of high concentrations of 5-fluorouracil in human and murine tumors as compared with plasma.. Cancer Chemother Pharmacol.

[OCR_00662] Peters G. J., Laurensse E. J., Leyva A., Pinedo H. M. (1986). Tissue homogenization using a micro-dismembrator for the measurement of enzyme activities.. Clin Chim Acta.

[OCR_00676] Peters G. J., van der Wilt C. L., van Groeningen C. J., Smid K., Meijer S., Pinedo H. M. (1994). Thymidylate synthase inhibition after administration of fluorouracil with or without leucovorin in colon cancer patients: implications for treatment with fluorouracil.. J Clin Oncol.

[OCR_00682] Plumb J. A., Gerritsen M., Milroy R., Thomson P., Workman P. (1994). Relative importance of DT-diaphorase and hypoxia in the bioactivation of EO9 by human lung tumor cell lines.. Int J Radiat Oncol Biol Phys.

[OCR_00692] Plumb J. A., Gerritsen M., Workman P. (1994). DT-diaphorase protects cells from the hypoxic cytotoxicity of indoloquinone EO9.. Br J Cancer.

[OCR_00686] Plumb J. A., Workman P. (1994). Unusually marked hypoxic sensitization to indoloquinone EO9 and mitomycin C in a human colon-tumour cell line that lacks DT-diaphorase activity.. Int J Cancer.

[OCR_00699] Prochaska H. J., Talalay P. (1988). Regulatory mechanisms of monofunctional and bifunctional anticarcinogenic enzyme inducers in murine liver.. Cancer Res.

[OCR_00702] Riley R. J., Workman P. (1992). DT-diaphorase and cancer chemotherapy.. Biochem Pharmacol.

[OCR_00714] Robertson N., Haigh A., Adams G. E., Stratford I. J. (1994). Factors affecting sensitivity to EO9 in rodent and human tumour cells in vitro: DT-diaphorase activity and hypoxia.. Eur J Cancer.

[OCR_00708] Robertson N., Stratford I. J., Houlbrook S., Carmichael J., Adams G. E. (1992). The sensitivity of human tumour cells to quinone bioreductive drugs: what role for DT-diaphorase?. Biochem Pharmacol.

[OCR_00718] Ross D., Siegel D., Beall H., Prakash A. S., Mulcahy R. T., Gibson N. W. (1993). DT-diaphorase in activation and detoxification of quinones. Bioreductive activation of mitomycin C.. Cancer Metastasis Rev.

[OCR_00724] Schellens J. H., Planting A. S., van Acker B. A., Loos W. J., de Boer-Dennert M., van der Burg M. E., Koier I., Krediet R. T., Stoter G., Verweij J. (1994). Phase I and pharmacologic study of the novel indoloquinone bioreductive alkylating cytotoxic drug E09.. J Natl Cancer Inst.

[OCR_00734] Schlager J. J., Powis G. (1990). Cytosolic NAD(P)H:(quinone-acceptor)oxidoreductase in human normal and tumor tissue: effects of cigarette smoking and alcohol.. Int J Cancer.

[OCR_00740] Schor N. A., Cornelisse C. J. (1983). Biochemical and quantitative histochemical study of reduced pyridine nucleotide dehydrogenation by human colonic carcinomas.. Cancer Res.

[OCR_00754] Siegel D., Beall H., Senekowitsch C., Kasai M., Arai H., Gibson N. W., Ross D. (1992). Bioreductive activation of mitomycin C by DT-diaphorase.. Biochemistry.

[OCR_00746] Siegel D., Gibson N. W., Preusch P. C., Ross D. (1990). Metabolism of diaziquone by NAD(P)H:(quinone acceptor) oxidoreductase (DT-diaphorase): role in diaziquone-induced DNA damage and cytotoxicity in human colon carcinoma cells.. Cancer Res.

[OCR_00758] Smitskamp-Wilms E., Peters G. J., Pinedo H. M., van Ark-Otte J., Giaccone G. (1994). Chemosensitivity to the indoloquinone EO9 is correlated with DT-diaphorase activity and its gene expression.. Biochem Pharmacol.

[OCR_00764] Stratford I. J., Adams G. E., Bremner J. C., Cole S., Edwards H. S., Robertson N., Wood P. J. (1994). Manipulation and exploitation of the tumour environment for therapeutic benefit.. Int J Radiat Biol.

[OCR_00770] Wallin R., Gebhardt O., Prydz H. (1978). NAD(P)H dehydrogenase and its role in the vitamin K (2-methyl-3-phytyl-1,4-naphthaquinone)-dependent carboxylation reaction.. Biochem J.

[OCR_00782] Walton M. I., Bibby M. C., Double J. A., Plumb J. A., Workman P. (1992). DT-diaphorase activity correlates with sensitivity to the indoloquinone EO9 in mouse and human colon carcinomas.. Eur J Cancer.

[OCR_00776] Walton M. I., Smith P. J., Workman P. (1991). The role of NAD(P)H: quinone reductase (EC 1.6.99.2, DT-diaphorase) in the reductive bioactivation of the novel indoloquinone antitumor agent EO9.. Cancer Commun.

[OCR_00788] Walton M. I., Sugget N., Workman P. (1992). The role of human and rodent DT-diaphorase in the reductive metabolism of hypoxic cell cytotoxins.. Int J Radiat Oncol Biol Phys.

[OCR_00808] Workman P., Binger M., Kooistra K. L. (1992). Pharmacokinetics, distribution, and metabolism of the novel bioreductive alkylating indoloquinone EO9 in rodents.. Int J Radiat Oncol Biol Phys.

[OCR_00804] Workman P. (1992). Bioreductive mechanisms.. Int J Radiat Oncol Biol Phys.

[OCR_00814] Yao K. S., Clayton M., O'Dwyer P. J. (1994). Interaction of heat and hypoxia in modulating transcription of DT diaphorase in human colon adenocarcinoma cells.. Cell Growth Differ.

